# Harnessing paleo‐environmental modeling and genetic data to predict intraspecific genetic structure

**DOI:** 10.1111/eva.12986

**Published:** 2020-06-02

**Authors:** Glenn Yannic, Oskar Hagen, Flurin Leugger, Dirk N. Karger, Loïc Pellissier

**Affiliations:** ^1^ Univ. Grenoble Alpes Univ. Savoie Mont Blanc CNRS LECA Grenoble France; ^2^ Landscape Ecology Department of Environmental Systems Sciensce Institute of Terrestrial Ecosystems ETH Zürich Zürich Switzerland; ^3^ Swiss Federal Institute for Forest, Snow and Landscape Research Birmensdorf Switzerland

**Keywords:** climate change, landscape genetics, migration, population genetics, range dynamics, refugia, species distribution modeling

## Abstract

Spatially explicit simulations of gene flow within complex landscapes could help forecast the responses of populations to global and anthropological changes. Simulating how past climate change shaped intraspecific genetic variation can provide a validation of models in anticipation of their use to predict future changes. We review simulation models that provide inferences on population genetic structure. Existing simulation models generally integrate complex demographic and genetic processes but are less focused on the landscape dynamics. In contrast to previous approaches integrating detailed demographic and genetic processes and only secondarily landscape dynamics, we present a model based on parsimonious biological mechanisms combining habitat suitability and cellular processes, applicable to complex landscapes. The simulation model takes as input (a) the species dispersal capacities as the main biological parameter, (b) the species habitat suitability, and (c) the landscape structure, modulating dispersal. Our model emphasizes the role of landscape features and their temporal dynamics in generating genetic differentiation among populations within species. We illustrate our model on caribou/reindeer populations sampled across the entire species distribution range in the Northern Hemisphere. We show that simulations over the past 21 kyr predict a population genetic structure that matches empirical data. This approach looking at the impact of historical landscape dynamics on intraspecific structure can be used to forecast population structure under climate change scenarios and evaluate how species range shifts might induce erosion of genetic variation within species.

## 1 UNDERSTANDING THE ORIGIN OF SPATIAL GENETIC DIVERSITY AND STRUCTURE

Global climate change is expected to pressure species to shift species ranges at an unprecedented rate potentially causing significant biodiversity decline (Johnson et al., 2017; Pacifici et al., 2015; Parmesan & Yohe, 2003; Pecl et al., 2017; Peñuelas et al., 2013; Ripple et al., 2015; Thomas et al., 2004; Urban, 2015). Because of limited dispersal, species might be unable to track their suitable habitat and get extinct (Årevall, Early, Estrada, Wennergren, & Eklöf, 2018). Anticipating the impacts of climate change is central to guide species conservation and management strategies as well as the design of protected areas (Dawson, Jackson, House, Prentice, & Mace, 2011; Jones, Watson, Possingham, & Klein, 2016; Keppel et al., 2012). Climate change is expected to erode not only species diversity within assemblages, but also intraspecific genetic variation (Scheffers et al., 2016), which is crucial for maintaining the ability of species to cope with new environmental conditions (Bijlsma & Loeschcke, 2012; Des Roches et al., 2018; Hoffmann & Sgrò, 2011; Hughes, Inouye, Johnson, Underwood, & Vellend, 2008; Razgour et al., 2019; Thuiller et al., 2011). Understanding how historical processes impacted intraspecific genetic structure and diversity can help anticipate ongoing and future global changes (Alsos et al., 2012; Davis & Shaw, 2001; Haywood et al., 2019; Yannic, Pellissier, Ortego, et al., 2014).

The climates of the glacial cycles of the Pleistocene, in particular the cold period of the last glacial maximum (LGM, 21 kyr BP; Clark et al., 2009), reshaped the distribution of ecosystems (Williams, & Jackson, 2007), species ranges (Hampe & Jump, 2011; Hewitt, 1999), and intraspecific genetic structure (Yannic, Pellissier, Ortego, et al., 2014). Climate dynamics and its control over the distribution of the continental ice sheet caused dramatic species range expansions and contractions in the Northern Hemisphere (Alsos et al., 2012). Species dispersal is a central determinants of range shifts, but also has consequences on the genetic structure within a species range (Davis & Shaw, 2001; Szűcs et al., 2017). In particular, repeated isolation of populations during successive glacial cycles has generated complex genetic differentiation within species (Hewitt, 2004; Hofreiter & Stewart, 2009; Lister, 2004). Moreover, genetic drift during population range shift and range contraction can erode the genetic diversity of populations, as theoretically predicted (Arenas, Ray, Currat, & Excoffier, 2011; Garnier & Lewis, 2016; McInerny, Turner, Wong, Travis, & Benton, 2009), empirically shown (Alsos et al., 2007) or forecasted under climate change (e.g., Collevatti, Nabout, & Diniz‐Filho, 2011). The current genetic structure of species is thus expected to be intimately related to the historical spatial and temporal variation in their distribution ranges, which in turn has shaped the pattern and frequency of population genetic exchanges or the degree of genetic differentiation (Espíndola et al., 2012; Pellissier et al., 2016).

Landscape genetics as a discipline integrates spatially explicit data to investigate the influence of landscape heterogeneity on contemporary gene flow (Balkenhol, Cushman, Storfer, & Waits, 2016). Landscape features and habitat characteristics can have a profound impact on genetic structure of populations, by either restricting or enhancing individual movements and populations connectivity (Taylor, Fahrig, Henein, & Merriam, 1993; Taylor, Fahrig, & With, 2006). A central tenet of landscape genetics is to identify patterns of suitable habitats or sets of features that promote or hinder connectivity among patches and shape the genetic structure of species (Zeller, McGarigal, & Whiteley, 2012). Landscape resistance to gene flow is parameterized using different approaches: (a) expert opinions (Murray et al., 2009), (b) optimization and parameterization methods (e.g., Peterman, 2018; Spear, Balkenhol, Fortin, McRae, & Scribner, 2010), or (c) occurrence, presence‐data, or satellite‐collar relocations coupled with species distribution modeling (Shafer et al., 2012; Yannic, Pellissier, Le Corre, et al., 2014; Zeller et al., 2018). The advantage of data‐based inferences in relation to environmental variables is that it allows constructing more objective models of habitat use and can identify corridors based on true locations or species’ preferred habitat (Fattebert, Robinson, Balme, Slotow, & Hunter, 2015; Panzacchi et al., 2016). Landscape feature shown to impact connectivity and genetic distinctiveness today could have had a similar effect in the past. Hence, the study of intraspecific genetic structure requires the identification of historical landscape elements that shaped gene flow through the analysis of paleo‐environmental maps coupled with species ecological information.

The climate distribution within landscapes was largely dynamic over the past millennia (Batchelor et al., 2019). Originally, the distributions of paleoclimate were reconstructed using indicators such as pollens, environmental DNA (eDNA), oxygen isotopes, or other features (Koch, 1998; Lyman, 2017; Parducci et al., 2017; Willerslev et al., 2007, 2014). The position of moraines was, for example, used for reconstructing the extent of glaciated area in the past millennia and its effect on landscape connectivity (Nesje, Bakke, Dahl, Lie, & Matthews, 2008). Fossilized indicators and eDNA from lake sediments were used for reconstructing past vegetation patterns and informed on species range segregation (Alsos et al., 2016). Vegetation reconstruction using fossil records showed that the tree line largely shifted during glacial periods (Binney et al., 2017; Payette & Lavoie, 1994), possibly shaping connectivity for other organisms. The development and refinement of global climate models (GCMs) provide another source for the reconstruction of past landscape dynamics (Haywood et al., 2019). The downscaling of GCMs from coarse to fine resolution can generate the changes in abiotic conditions that constrain the distribution of organisms over time (Latombe et al., 2018). Combining different sources for reconstructing past species range dynamics can help understand how past dynamics shaped present intraspecific genetic structure of species (Fordham et al., 2016; Fordham, Brook, Moritz, & Nogués‐Bravo, 2014; Gavin et al., 2014).

Beyond current landscape configuration, the genetic structure of populations is the result of an intermingling of past climatic effects, geography, and anthropogenic pressures on species (e.g., Lorenzen et al., 2011). Disentangling effects of different drivers on the current genetic structure of populations is however challenging and requires the use of integrative modeling tools (Epperson et al., 2010; Landguth, Cushman, & Balkenhol, 2015). We present an overview of the simulation approaches that were used to explore the determinants of extant genetic diversity and structure. We further illustrate a new modeling approach, conceptually simpler, which provides an expectation of intraspecific genetic structure as a result of the dynamic landscape suitability and connectivity for species.

## 2 INTEGRATING SIMULATIONS AND MODELING IN A GLOBAL LANDSCAPE GENETIC APPROACH

Genetic data, increasingly available (e.g., Lorenzen et al., 2011), can be used to infer past demographic history of species (a) directly, using genetic models, in the light of geographic information data, or (b) indirectly, using process‐based spatial simulation models (e.g., Campos et al., 2010; Drummond, Rambaut, Shapiro, & Pybus, 2005; Shapiro et al., 2004). Moreover, advanced analyses from genomic data can support the quantification of past demographic events such as estimations of past population sizes or the occurrence of demographic bottlenecks (Hansen et al., 2018; Nadachowska‐Brzyska, Li, Smeds, Zhang, & Ellegren, 2015; Pilot et al., 2014; Stoffel et al., 2018). Through the comparison of spatially structured populations, genetic analyses can inform about ancestral population connectivity (e.g., temporal changes in the level of isolation‐by‐distance among ancient DNA samples; Lorenzen et al., 2011) and demographic history such as genetic bottlenecks driven by population disconnection (Broquet et al., 2010).

A variety of spatial data can be used to investigate the origin of intraspecific genetic structure of species, which include contemporary landscape descriptors (Sork et al., 2013; Storfer et al., 2007) and historical species distribution reconstruction (Nogués‐Bravo, 2009). Various landscape habitat characteristics are expected to influence the connectivity for populations and determine their genetic structure (Balkenhol et al., 2016). Methods to measure connectivity using cost‐weighted distance allowed refined quantification of landscape barriers to gene flow (Balkenhol et al., 2016). Another complementary spatial information is provided by species distribution models (SDMs), which by estimating species habitat suitability, can help understand the historical dynamics that shaped intraspecific genetic structure of populations (Carnaval, Hickerson, Haddad, Rodrigues, & Moritz, 2009; Lorenzen et al., 2011; Razgour et al., 2013; Yannic, Pellissier, Ortego, et al., 2014). Hindcasted SDMs were used to identify the geographic position of refugia (but see Davis, McGuire, & Orcutt, 2014), and assess visually if they match a similar structure found in the geographic distribution of genetic clusters (e.g., Waltari et al., 2007). The availability of more temporal steps for climate reconstructions between the LGM and the present allowed tracking climatic suitability area over time (Hijmans, Cameron, Parra, Jones, & Jarvis, 2005; Karger et al., 2017) and combined with simple dispersal models (Engler & Guisan, 2009), following the geographic range dynamics of isolated populations (Espíndola et al., 2012; Nobis & Normand, 2014). For example, Yannic, Pellissier, Ortego, et al. (2014) found two main refugia for the caribou/reindeer *Rangifer tarandus*, which explain current structure, whose dynamic until the present matches the current genetic structure of the species. Habitat suitability values can be used in more complex process‐based model to simulate intraspecific genetic structure in forward‐going simulations (Hoban, Bertorelle, & Gaggiotti, 2012).

The adoption of spatial models including genetic mechanisms can inform the process that shapes current intraspecific genetic diversity and structure (Landguth et al., 2015). Generally, the best available confirmation of the understanding of the causes of a phenomenon is the ability to build a model from the expected underlying mechanisms and reproduce realistic emergent patterns (Leprieur et al., 2016). Process‐based models place causal hypotheses on a leading role, as an a priori abstraction of the inner workings of the system that is used to build the model (Grimm et al., 2005). More specifically, process‐based models in landscape genetics are primarily built to evaluate analytical issues, *for example*, the effects of spatial and temporal scale in landscape genetics (Jaquiéry, Broquet, Hirzel, Yearsley, & Perrin, 2011), or to investigate theoretical questions, *for example*, quantifying the lag time between the emergence of a barrier to movement and its effects on spatial genetic data (Landguth et al., 2010). The expectation of those models can be then compared to empirical data qualitatively and quantitatively (Jeltsch et al., 2013; Landguth et al., 2015). Based on a limited number of principles and in combination with the reconstructed dynamics of landscape suitability for species, process‐based models are able to produce expectations related to intraspecific genetic diversity and structure that matches empirical data (Landguth et al., 2015). A variety of spatial process‐based models of species genetics have been developed and include a variety of processes (Table 1; Box 2), such as genetic drift (*CDPop*; Landguth & Cushman, 2010), geographic isolation (*IBDSim*; Leblois, Estoup, & Streiff, 2006), selection, and adaptation (*Nemo*; Guillaume & Rougemont, 2006). The importance of simulation studies for this specific research has been emphasized in a number of recent articles (e.g., Balkenhol, Waits, & Dezzani, 2009; Epperson et al., 2010).

**TABLE 1:** Detailed information on spatially explicit landscape genetics simulators. We provide information on (a) the type of simulators between forwards‐in‐time simulations (also known as individual‐based simulations) and backwards‐in‐time simulations (also known as coalescent simulations) and (b) the level of program between individual‐based and population‐based simulations. We also provide general descriptions of programs based on direct citations from original journal articles

Program	Simulators	Level	Description	Ref.
Splatche 3	Backward	Population	Simulate the demography of populations and the genetic data (e.g., DNA sequences, SNPs, STRs) are simulated under a serial coalescent‐based approach. The simulation framework is spatially explicit and accounts for the heterogeneity and the dynamic of landscapes. see also Aquasplatche (Neuenschwander 2006), extension of Splatche for river habitats.	Currat, Arenas, Quilodràn, Excoffier, and Ray (2019)
PhyloGeoSim	Backward	Population	Simulates the evolution of DNA sequences under a model of coalescence on a twodimensional grid. Populations can exchange gene copies and/or host coalescence events between two or more gene copies. Simulate data under evolutionary scenarios taking both demographic and geographic characteristics into account (e.g., isolation by distance, fragmentation, expansion, secondary contact).	Dellicour, Kastally, Hardy, and Mardulyn (2014)
IBDSim	Backward	Population	Simulate genetic data under isolation by distance. Several dispersal distributions are considered. Applied to test the effect of various sampling, mutational, and demographic factors on the pattern of genetic variation.	Leblois, Estoup, and Rousset (2009)
Quetzal	Backward	Population	Implement a wide range of coalescence‐based environmental demogenetic models. Using available genetic samples, aims to understand and foresee the behavior of species living in a fragmented and temporally changing environment. Include a model of coalescence conditioned to environment, through an explicit modeling of population growth and migration. Parameters can be inferred using Approximate Bayesian Computation.	Becheler, Coron, and Dupas (2019)
cdpop	Forward	Individual	Simulate gene flow in complex landscapes; model gene flow among spatially located individuals as a function of individual‐based movement through mating and dispersal, incorporating population dynamics and evolutionary forces that affect allele frequency (mutation, gene flow, genetic drift, and selection).	Landguth et al. (2010)
Nemo	Forward	Individual	Simulate evolution of life history/phenotypic traits and population genetics in a (meta‐) population framework. Allows for patch‐specific carrying capacities, dispersal rates, stochastic extinction/harvesting rates, and demographic stochasticity. Allow population bottlenecks, patch fusion/fission, population expansion, etc. during the simulation process.	Guillaume and Rougemont (2006)
CDMetaPOP	Forward	Individual	Simulate landscape demographic and genetics (defined herein as landscape demogenetics). Simulate changes in neutral and/or selection‐driven genotypes through time as a function of individual‐based movement, complex spatial population dynamics, and multiple and changing landscape drivers.	Landguth, Bearlin, Day, and Dunham (2017)
popRange	Forward	Individual	Simulate spatially and temporally explicit scenarios with chromosome‐scale data. Features such as spatially and temporally variable selection coefficients are incorporated in a flexible manner. This software allows for large‐scale analyses and comparisons of these complex, stochastic models.	McManus (2015)
SimAdapt	Forward	Individual	A spatially explicit, individual‐based, forward‐time, landscape‐genetic simulation model, combined with a landscape cellular automaton to represent evolutionary processes of adaptation and population dynamics in changing landscapes, using the NetLogo environment.	Rebaudo, et al. (2013)
EcoGenetics	Forward	Individual	Simulate spatially explicit metapopulations. Allows defining all kinds of landscape configurations, from real landscapes (e.g., stemming from Geographic Information Systems) to theoretical structures (e.g., island model, circles, grid). Consider various life history, including sex‐dependent or age‐dependent behaviors and generation overlap	Jaquiéry, et al., (2011)
SLiM	Forward	Population	Simulation framework that combines a powerful, fast engine for forward population genetic simulations with the capability of modeling a wide variety of complex evolutionary scenarios. Supports models that occupy continuous spatial landscapes, including built‐in support for spatial maps that describe environmental characteristics. Possible to model the explicit movement of individuals over a continuous landscape, life cycles with overlapping generations, individual variation in reproduction, density‐dependent population regulation, individual variation in dispersal or migration, local extinction and recolonization, mating between subpopulations, age structure, fitness‐based survival and hard selection, emergent sex ratios, and more.	Haller and Messer (2019)
quantiNemo	Forward and backward	Individual	Individual‐based, genetically explicit stochastic simulation program. Developed to investigate the effects of selection, mutation, recombination, and drift on quantitative traits in structured populations connected via migration in a heterogeneous landscape	Neuenschwander, Michaud, and Goudet (2018)
MEMGENE	Sideway	Population	Method and software for identifying spatial neighborhoods in genetic distance data. Use a multivariate technique developed for spatial ecological analyses. Using simulated genetic data, allow recover patterns reflecting the landscape features that influenced gene flow.	Galpern, Peres‐Neto, Polfus, and Manseau (2014)

Box 1 **Scientific curiosity and expertise know no boundaries**

This word cloud was picked up on the web page of Louis Bernatchez (Figure Box 1). This figure illustrated the main research topics of Louis’ laboratory, *that is*, fish and genes. It would however be restrictive to resume the Louis’ research activities to the population genetics and genomics of fishes, and more generally, of freshwater and marine organisms, as highlighted by some seminal papers not directly related to fishes, *for example*, on MHC in nonmodel vertebrates (Bernatchez & Landry, 2003) or on the definition of conservation units (Fraser & Bernatchez, 2001). Louis also regularly hosts in his laboratory some outliers. I was one of them, working mainly on questions related to mammal and bird population genetics (e.g., in collaboration with Louis on black bear *Ursus americanus* (Roy, Yannic, Côté, & Bernatchez, 2012) or caribou (Yannic, Pellissier, Ortego, et al., 2014; Yannic et al. 2016)). My few years as a postdoc in Louis' laboratory in early 2010 gave me a glimpse into the world of “omics,” and since marked my progressive shift from population genetics to population genomics. All along these years, I was really impressed by the stimulating and friendly atmosphere within the Louis Bernatchez’ laboratory, the easiness of people to share experience, codes, laboratory tips, and to work collaboratively. In the same vein, stimulated by Louis, people were always aware of the most recent scientific literature, sharing papers and ideas. During my time in Quebec City, I also remember (“Je me souviens”) the use of the cutting‐edge molecular biology technologies and methods to answer theoretical questions in evolutionary biology, but which often have strong implications for applied science. One of the strengths of Louis’ work is to build bridges between a high‐level academic research and conservation and management programs. Scientific curiosity and expertise know no boundaries.

**FIGURE B1:**
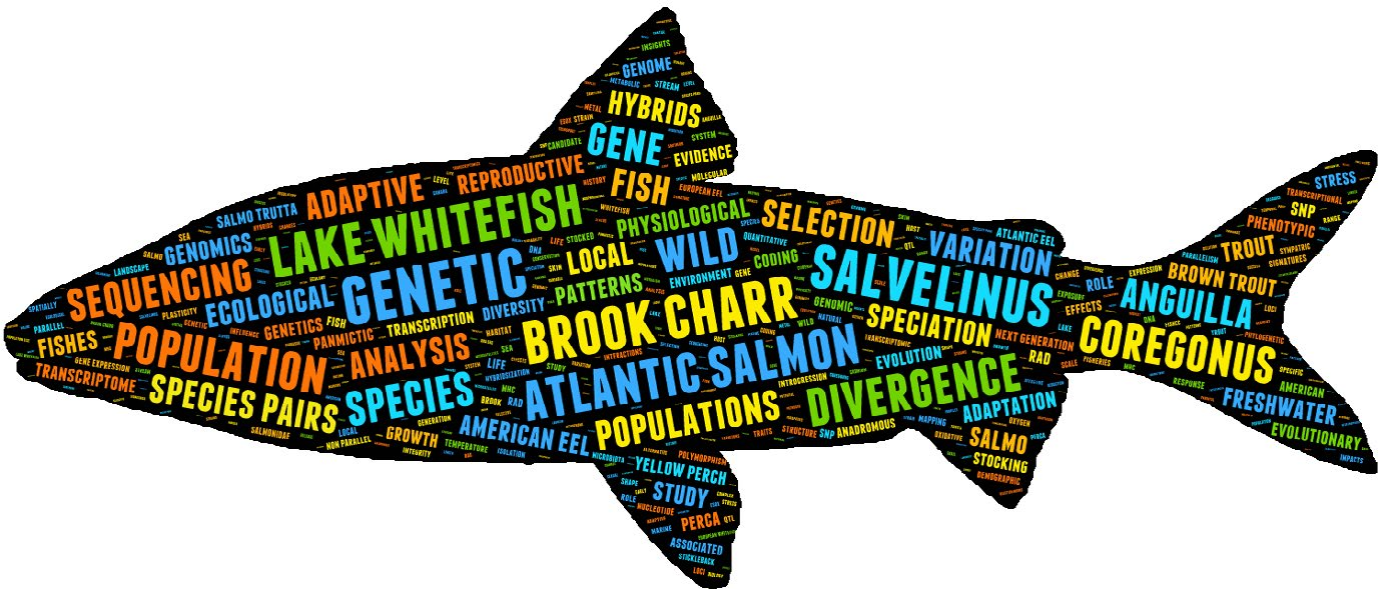
Word cloud of Louis Bernatchez’ s research interests © Louis Bernatche (http://www2.bio.ulaval.ca/louisbernatchez/presentation.htm)

Box 2 **A geographic model of genetic beta diversity**

Process‐based models of intraspecific genetic diversity and structure represent a simplification of the reality, and their development is generally done along three main lines of abstraction: (a) the nature of the modeled agent (e.g., individuals or populations), (b) the detail of the mechanisms (e.g., drift, selection), and (c) the characterization of the landscape dynamic. A first line of abstraction is the characterization of the agent, which might depend on the mechanisms and the type of emergent pattern (e.g., population genetic diversity, *F*_ST_) that the model should explore. The definition of the agent, over which mechanisms are acting and whose interactions with the landscape generate emergent genetic patterns is crucial. Depending on the research questions, the agent can be (a) genes (e.g., Guillaume & Rougemont, 2006), (b) individuals (e.g., Melián, Seehausen, Eguíluz, Fortuna, & Deiner, 2015), or (c) populations (e.g., Rangel et al., 2018). A second line of abstraction is the implementation of processes acting on agents, ranging from genetic drift (e.g., *CDPop*), mutation (e.g., *EcoGenetics*), recombination (e.g., *quantiNemo*), or hybridization (e.g., *PhyloGeoSim*, *Splatche*; see Table 1 for details on simulation models). A third line of abstraction is the numerical simplification of the landscape over which agents and mechanisms play out. The landscape can be modeled with more or less complexity, for instance how different features (*e.g*., water, forested areas) influence the permeability to the dispersal of a species or using the outputs of SDMs.

Simulation studies require simplifying assumptions tailored to the specific research question, and those should correspond to the foreseen comparison to empirical data (Landguth et al., 2015). In order to study the variety of empirical patterns, relying on a unique general modeling approach is unrealistic and a diversity of more specific model implementations should be preferred (Landguth et al., 2015). Specific models could be implemented in agreement with the research question and the property of interest (Landguth et al., 2015). A model generally represents a simplification of the complexity of natural processes and can be developed to specifically investigate one or a limited number of specific properties of a system (e.g., neutral genetic diversity rather than genetic structure, Grimm et al., 2005). The implementation of a model depends on very specific assumptions that are made when translating biological processes into a computer algorithm (Grimm & Railsback, 2012). The complexity of the models largely depends on data availability (e.g., species dispersal capacity, population demographic parameters). Hence, even when investigating a specific empirical property, it is recommended to rely on models developed independently. As a result, understanding the spatial variability in population genetic data can be tackled from different modeling angles.

**FIGURE 1:**
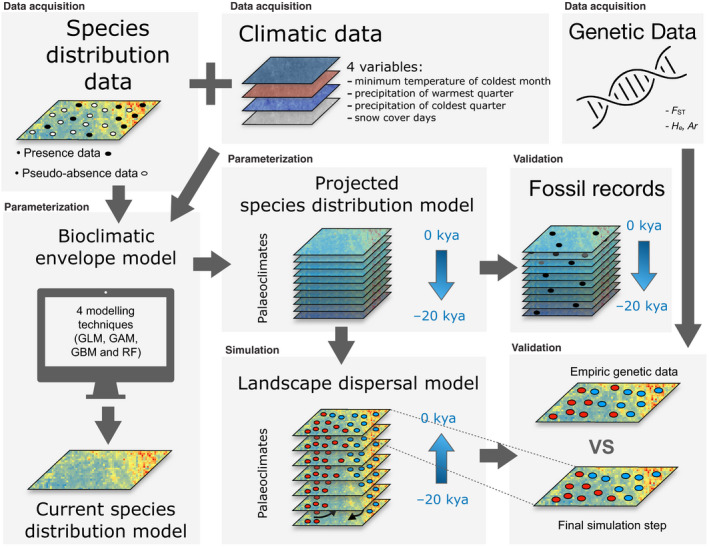
Workflow delineating the main steps used in our integrative approach harnessing current and past species distribution modeling, landscape genetics simulation, empirical genetic, and fossil data in order to identify the drivers that shape the current intraspecific genetic diversity

We present a parsimonious model allowing inference on population genetic structure given the suitability of species occupancy of the landscape, the species dispersal capacity, and the landscape structure shaping connectivity among cells (Figure 1, Box 2). In contrast to previous approaches focused on the demographic processes that are shaping the genetic diversity within populations (or genetic alpha diversity), this modeling approach focuses on how landscape dynamics generate differentiation between populations (or genetic beta diversity) over time. We adopt a parsimony principle, where the model is conceptually simple as the genetic processes are not modeled explicitly. The model can integrate the effect of various landscape features on population connectivity and work at high spatial resolution. The model includes a limited number of parameters, where dispersal is central to generate simulated population isolation. Dispersal determines populations spatial clustering in interaction with the landscape features via the computation of connectivity matrices (Balkenhol et al., 2016). The computation of the connectivity matrix is as important as the suitability matrix itself to determine species dynamics and the connectivity of populations. Features of the environment can be included to determine whether they act as barriers or as corridors for species dispersal, and rely on the computation of matrices as classically performed in landscape ecology (Balkenhol et al., 2016; Sork et al., 2013; Storfer et al., 2007). Our framework is presented on Figure 1. We illustrate our approach using a previously published dataset on the case study of reindeer/caribou across North America and Eurasia (Yannic et al., 2018; Yannic, Pellissier, Ortego, et al., 2014).

## 3 METHODS

### 3.1 Genetic data

**FIGURE 2:**
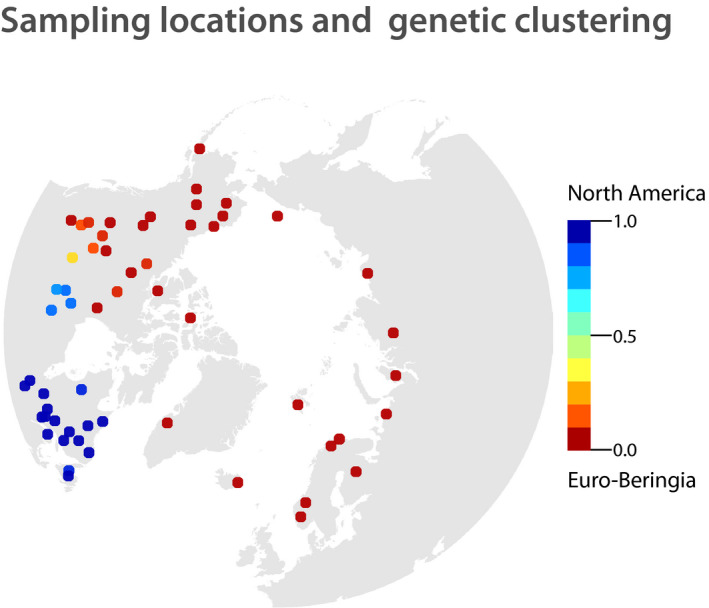
Distribution of caribou and reindeer *Rangifer tarandus* sampled herds across the Holarctic distribution of the species. Sampling locations are colored according to population membership of each herd to the North American clade, considering two genetic clusters (from blue for North American clade to red for Euro‐Beringian clade) as inferred in Yannic, Pellissier, Ortego, et al. (2014). For more details on sampling locations, see Yannic, Pellissier, Ortego, et al. (2014); Yannic et al., 2018)

We used a set of 1,297 caribou and reindeer genotyped at 16 nuclear microsatellite markers (Yannic et al., 2018; Yannic, Pellissier, Ortego, et al., 2014). Samples were obtained from 57 locations located across the entire Holarctic native species’ range, including Alaska, Canada, Greenland, Svalbard, Norway, Finland, and the Russian Federation (Figure 2). Samples were collected over a decade (2000–2010). We tried to cover as much as possible the known circumpolar distribution of the species, although some areas are not evenly represented (e.g., few populations from Russia versus numerous herds from Canada; Figure 2). In these previous works, using multivariate and Bayesian analysis, we identified that the genetic structure of *Rangifer* was split into two main phylogeographic clusters at the uppermost hierarchical level (Yannic et al., 2018; Yannic, Pellissier, Ortego, et al., 2014). Using climatic niche modeling, we further identified refugia occupied by the two lineages at the LGM (i.e., 21 kyr BP), as defined by discontinuous suitability for the species, *that is*, suitable areas located south and northwest of the Laurentide Ice Sheet in North America (see panel “21 ka” in Figure 2; Yannic, Pellissier, Ortego, et al., 2014). We finally used a dispersal model (Engler & Guisan, 2009) to track the species range shift overtime (between 21 kyr BP to present) from these distinct refugia that lead to the current observed genetic structure of *Rangifer* populations.

### 3.2 Paleoclimate

We created a centennial time series for the times from 21’000 BP to 1990 using a variant of the CHELSA V1.2 algorithm (Karger et al., 2017) on the TraCE‐21ka data (Liu et al., 2009) (hereafter: CHELSA‐TraCE). The TraCE‐21ka dataset contains monthly atmospheric output from transient simulations of a fully coupled, nonaccelerated atmosphere‐ocean‐sea ice‐land surface simulation using CCSM3 and starting at the LGM to present. The simulation starts at 22,000 years before present (22 kyr BP) and finishes in 1990 CE. TraCE‐21ka runs at a T31 grid resolution with 96 by 48 horizontal grid cells (approximately 3.75°) resolution, which is too coarse for ecological inference. We downscaled the TraCE‐21ka data to 30‐arc second resolutions by complementing the CHELSA V1.2 algorithm with a temperature coupled ice sheet interpolation using ICE 6G (Peltier, Argus, & Drummond, 2015) to estimate the potential orography at 100‐year time steps. Based on this orography, we calculated mean monthly precipitation rates, mean monthly daily maximum, and mean monthly daily minimum temperatures, bioclimatic variables, and snow cover using the methods described in Karger et al. (2017) and Karger et al. (2019).

### 3.3 Species distribution model

Species distribution models were calibrated using ensembles of four statistical techniques: generalized linear models (GLM), generalized additive models (GAM) with the *gam* R package (Hastie, 2019), gradient boosting machine (GBM) with the *gbm* R package (Greenwell, Boehmke, Cunningham, & Developers, 2019), and RandomForest (RF) with the *randomForest* R package (Liaw & Wiener, 2002). We randomly sampled 3,000 pseudo‐absences on the Northern Hemisphere from 20 to 84 degrees north at 1° resolution (Barbet‐Massin, Jiguet, Albert, & Thuiller, 2012). We rasterized the IUCN range map for the caribou (Gunn, 2016) at 1° and randomly sampled 1’500 presences. We weighed the presences to provide an equal weight to pseudo‐absences. Four climatic variables from CHELSA‐TraCE (Karger et al., 2017) were used: (a) minimum temperature of the coldest month, precipitation of the (b) coldest and (c) warmest quarter, and (d) the snow cover days (average number of days per year where a cell is covered with snow). We evaluated the model performance using the area under the ROC plot curve (AUC), Kappa, and true skill statistics (TSS). We used a split sample approach (70% calibration data and 30% evaluation data) with 20 repetitions. Models are considered to have a reliable performance with AUC scores > 0.7 and TSS values > 0.4 (Allouche, Tsoar, & Kadmon, 2006; Guisan, Thuiller, & Zimmermann, 2017). We applied a binary classification of the suitability from each model output using the maximum TSS approach to define the threshold with the *presenceAbsence* R package. Areas covered by glaciers (Clark et al., 2009; Clark, 2012) were classified as nonsuitable habitat. To apply binary classification to the ensembles, we classified cells with predictions of at least two models as suitable climate. We used the threshold maximizing the TSS value to convert occurrence probabilities in presences/absences from the *PresenceAbsence* R package (Freeman & Moisen, 2008). The distribution was predicted with 100‐year time steps till the LGM, 20,000 years BP. We validated the projection to the past with dated fossil records from Matthew T. Boulanger (Pers. Comm.), Lorenzen et al. (2011), Sommer, Kalbe, Ekström, Benecke, and Liljegren (2014), and all references therein.

### 3.4 Computation of landscape connectivity through time

We precomputed matrices of connectivity between all the cells that are suitable for the species for all 100‐year time steps from the LGM to the present. Cost distance and electric circuit are common tool in landscape genetics to investigate the connectivity among populations (McRae, Dickson, Keitt, & Shah, 2008) and can be directly compared to genetic distance metrics such as *F*_ST_, chord distance, or distance‐based principal components analysis (see Shirk, Landguth, & Cushman, 2017 for an overview). We computed connectivity matrices based on cost functions but generalized the computation to multiple time steps from the LGM to the present. The connectivity matrix computation is based on the *gdistance* R package (van Etten, 2018) and can accommodate various dispersal costs of the different features of the landscape. Nonsuitable habitat increases the cost of crossing the particular cell(s) according to their classification (*e.g*., glacier or sea) and thus decreases the possible geographic distance over which a population can disperse. A population can disperse to another cell as long as the least cost path between them is cheaper than the dispersal distance of that population. We prepared matrices with different costs: Crossing unsuitable habitat and glaciers had a cost factor of 1.1 to 1.25, and crossing the (frozen) sea had a cost factor of 1.1 to 3.

### 3.5 Simulating differentiation among populations through time

The model components are defined as the following steps. *Step 1:* Habitat suitability change: The new binary classification of the SDMs discriminating suitable habitat from nonsuitable habitat is loaded. *Step 2:* A “genetic” divergence matrix between each population in each cell records the accumulation of differentiations (*D*) among occupied cells over time. Two populations are connected if the species dispersal capability (*d*) is higher than the least cost path between them. Disconnected populations accumulate differentiation by drift, while connected one loss genetic differentiation over time by gene flow. After preliminary analyses (data not shown), we found that during the colonization process, genetic differentiation by drift occurred faster than genetic homogenization by gene flow. Therefore, we set the accumulation of divergence to occur three times as fast as the reduction. *Step 3:* Dispersal allows gene flow among connected cells at a distance *d* as presented above, but further allows the colonization of uncolonized cells. Species can disperse to all connected cells, which have a least cost path less than the dispersal distance *d*. The cells occupied by the species will change over time together with the genetic distance matrix quantifying pairwise differentiation between all the occupied cells.

As a burn‐in at the start of the simulation, we replicated the oldest time step (20,000 years BP) for 50 time steps to accumulate genetic differentiations between the isolated populations before the dynamic simulation starts. As parameters in the illustration simulations, we randomly draw dispersal distances for each cell from a Weibull distribution with shapes of 1 and 2.5 with median dispersal ranges from 2.1 km to 26 km per year (95% quantile ranging from 4.7 to 89 km/year). Values were selected according to the reported changes in calf ground location which reach a maximum speed of 15 km/year (Taillon, Festa‐Bianchet, & Côté, 2012) to 25 km/year (Couturier, Jean, Otto, & Rivard, 2004) over nearly 20 years. Together with the sets of distance matrices, we run in total 70 simulations exploring different dispersal and landscape connectivity parameters combinations.

### 3.6 Comparison between model and genetic data

We applied a principal coordinate analysis (PCoA) on the pairwise distance matrices obtained from our model as well as the Cavalli‐Sforza chord distance *Dc* (Cavalli‐Sforza & Edwards, 1967) and *F*_ST_ value from 1,297 caribou individuals sampled from 57 locations around their circumpolar distribution (Yannic et al., 2018; Yannic, Pellissier, Ortego, et al., 2014). The PCoA was computed with the *vegan* R package (Oksanen et al., 2019) and the phylogenetic trees with the *ape* R package (Paradis, Claude, & Strimmer, 2004; Paradis & Schliep, 2018). We mapped the resulting ordinated values and compared simulated ordination and empirical data. We also compared simulated and empirical pairwise genetic distance matrices using Spearman's correlation, and significance was tested with Mantel tests implemented in the *vegan* R package. Because not all currently viable populations have been predicted with the model (and vice versa), we removed all populations which are not included in both the model and the genetic dataset for the comparison (see Results). We applied a buffer of 200 km around the genetic sampling location to assign the location to the simulation cluster.

## 4 RESULTS

### 4.1 Species distribution models

**FIGURE 3:**
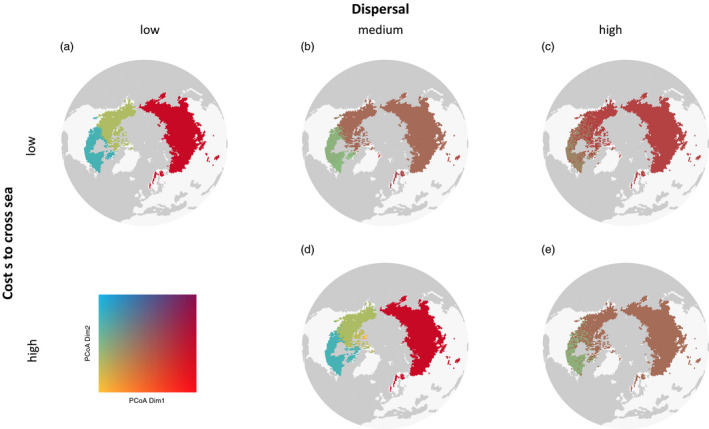
Simulated present genetic distinctiveness for five different simulation parameter settings of dispersal and landscape costs. Upper row (a)‐(c): costs for crossing water equal costs for crossing glaciers. They are increased by a factor of 5/4 compared to suitable habitat. Lower row (d)‐(e): costs for crossing water are increased by a factor of 10/3 compared to suitable habitat and costs for glaciers by 5/4. The dispersal distances are increasing from left to right; 1st column (a): median dispersal distance (mdd): 2.5km/year (95% quantile: 4.7km/year); 2nd column (b)‐(d): mdd: 4.3km/year (95%: 7.8km/year); 3rd column (c)‐(e): mdd 17km/year (95%: 31km/year). (b) Exhibits the output matching mostly to the observed genetic structure. Increasing the dispersal distance leads to only two genetic clusters, while increasing clearly the costs for crossing water results in two distinct populations on the side of the Bering Strait. The difference between (c) and (e) both with a high dispersal rate, but with a lower cost to cross the sea for (c) in comparison with (e), results in lower genetic difference between the Eurasia‐Beringia population and the one from northeastern America

The AUC value for the averaged model ensemble was 0.81 (GLM: 0.82, GAM: 0.83, GBM: 0.83, RF: 0.78), the Kappa value 0.49 (GLM: 0.48, GAM: 0.49, GBM: 0.5, RF: 0.47), and the TSS 0.58 (GLM: 0.56, GAM: 0.57, GBM: 0.59, RF: 0.58). We observed a large congruence between the projections of the four different SDMs. On average, 71% of cells, which were classified as suitable habitat, were predicted by all models, 87% by at least three and 95% by at least two models. The congruence between the models generally decreased over time. The average congruence between all four models between the present and 5’000 years BP was 76%, while it decreased to 64% between 15,000 years BP to 20,000 years BP. All SDMs showed two distinct refugia in northern America during the LGM. The northeastern refugia located in Alaska close to the Bering Strait and the southern one from the plains in the Midwest toward the Atlantic (see also Figure 3). Fifty out of 57 (88%) sampling locations were predicted as suitable habitat by the models (incl. 200‐km buffer; 45/57, 79%, with a 100km buffer).

### 4.2 Genetic simulation results

#### 4.2.1 Formation of the clusters. Effect of the cost distance

The cost distance for different habitat types strongly influenced the formation of population clusters (Figure 3). Simulations with lower costs for nonsuitable habitat showed more limited population structure in Northern America when the dispersal capacity was high or disconnected populations between Russia and Eastern North America in case of very low dispersal capacities. Exponential dispersal (Weibull shape of 1) resulted in events of long‐distance dispersal, which largely reduced the genetic structure among populations. The landscape and the dispersal parameters modulate the simulation outputs, which generate alternative expectations for the present genetic structure among populations that can be compared to the data.

#### 4.2.2 Comparison of the simulations with the data

**FIGURE 4:**
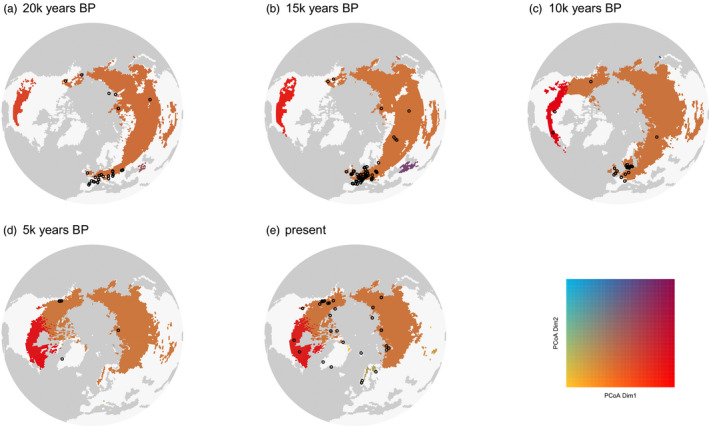
Simulated genetic distinctiveness among populations of caribou (*Rangifer tarandus*) from (a) 20,000 years BP, (b) 15,000 years BP, (c) 10,000 years BP, (d) 5,000 years BP, and (e) to the present. The parameters used in this simulation correspond to the one of Figure 3b): Median dispersal distance per year is 4.3 km, costs for crossing water equal cost for crossing unsuitable habitat. They are increased by a factor of 5/4 compared to suitable habitat. Black crosses represent fossil records in the interval [−1,000, +1,000] years around the time step, except for the present time step where the interval corresponds to [−1,000, 0] years. The habitable cells are defined as suitable from at least two out of four SDMs. The colors indicate the genetic distinctiveness of the populations/clusters according to their position on the first two axes of the PCoA (f) based on our model to simulate genetic distances. PCoA dimension one explains > 90% of the variance in all the examples shown. PCoA, principal coordinate analysis; SDM, species distribution models

**FIGURE 5:**
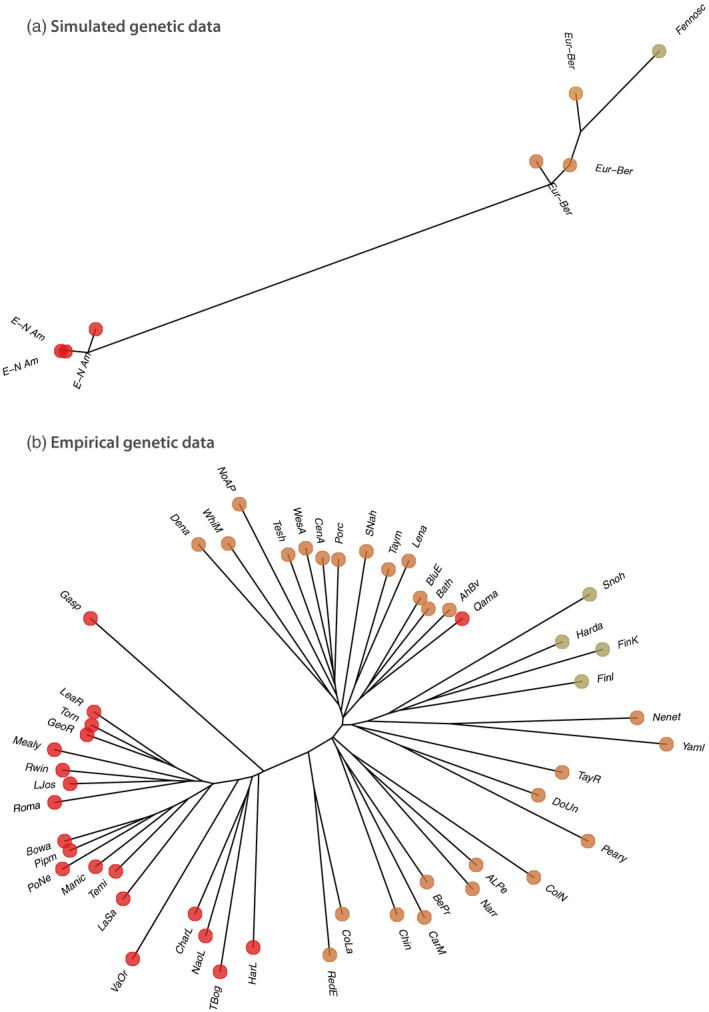
Dendrogram of the genetic structure recovered (a) from the model (left) and (b) from empirical data (right). The points are colored according to their location on Figure 4 (time series), and gray dots represent populations, which are not predicted in our model. We used pairwise chord distance to create the tree from the empirical data

**FIGURE 6:**
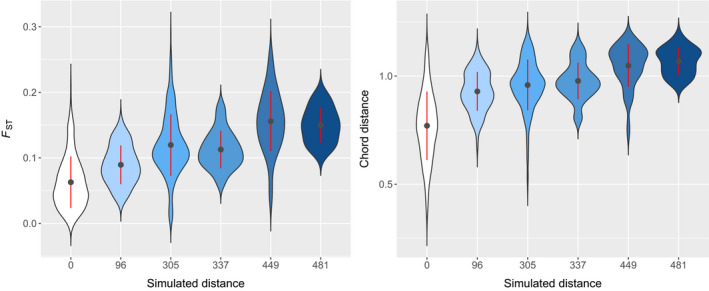
Violin plots depicting the correlation between pairwise empirical (*F*_ST_ and Chord distance) and simulated genetic distances. According to simulations, all populations that belong to the same cluster have a genetic distance equal to zero. Red dots correspond to mean values and error bars to standard deviation

Simulation outputs can be displayed in the form of genetic dendrograms and the corresponding distribution maps of the genetic clusters through time (Figures 4 and 5). The correlation between the pairwise genetic distances predicted by the best simulated model and the empirical genetic distances estimated with Chord distance is highly significant (Spearman's correlation = 0.65; Mantel's test *p*‐value < .001; *R* ^2^: 0.34, *p*‐value < .001) as well as with *F*_ST_ (Spearman's correlation = 0.59; Mantel's test *p*‐value < .001; *R* ^2^: 0.40, *p*‐value < .001; Figure 6). According to the SDMs and the simulations, the isolation during the glaciation formed two distinct clusters south of the North American ice sheet and in Beringia‐Siberia. Therefrom, the southern population started to expand its range northwards about 16,000 years ago, while the Beringian population dispersed southeastwards. The two populations became connected around 11,000 years BP. This formed a contact zone between the two genetically differentiated groups.

## 5 DISCUSSION

We provide a parsimonious method to simulate intraspecific genetic structure over a temporally dynamic landscape, which can be compared to empirical data. As a proof of concept, our illustrative application show the formation of two genetic clusters in the reindeer/caribou associated with two main different refugia during the LGM 20 kyr BP, *that is*, in Eurasia and central North America south of the ice sheet (see also Yannic, Pellissier, Ortego, et al., 2014). The simulation reproduces their dynamics from the LGM to the present shaping the extant genetic differentiation among populations (Figure 4). The simulation resulted in an intraspecific structure with two main clusters, each divided into subclusters, which corresponds to the observed genetic differentiation observed in the sampled populations (Figure 5). The SDMs provide the main constraints on the climatic suitability for a species from the LGM to the present, but the connectivity among suitable habitat patches further shapes the genetic structure within species. A broad parameter exploration allows evaluating the influence of landscape features and dispersal on the resulting genetic structure, where the comparison to the data informs on the biology of the species.

The approach developed here combining SDMs, paleo‐reconstructions, and simulations of genetic differentiation can be used to investigate the role of landscape features and dispersal ability on present intraspecific genetic structure of species. Intraspecific genetic structure largely depends on the level of gene flow among populations, which is shaped by species and landscape factors (Balkenhol et al., 2016). First, species display variation in their dispersal kernel defined as a probability density function describing the spread of individuals (Rogers et al., 2019), which can be explored using the dispersal kernel parameters. Caribou exhibit the longest terrestrial migrations on the planet, with multiple populations migrating over >1,200 km straight‐line, round‐trip distance and individuals moving as much as >4,800 km/year (Joly et al., 2019). This species is generally not dispersal limited except with long water barriers (Jenkins et al., 2016; Leblond, St‐Laurent, & Côté, 2016). In simulations, the largest dispersal distance explored best corresponded to the empirical data and agrees with measures of calf ground displacement, *that is*, 15–25 km/year (Couturier et al., 2004; Taillon et al., 2012). Second, properties of the landscape facilitate or hinder the movement of species and can further influence the gene flow among populations. Reconstructing vegetation types in the past might have provided more structure for the formation of genetic differentiation within each of the genetic clusters.

The levels of details in the simulations outputs depend on the complexity of the landscape features that are accounted for in the computation as well as the resolution of the SDMs. In our illustration, the simulated genetic structure showed more limited complexity that in empirical data (Figure 5). For instance, the lineage that had a refugia South of the North American ice sheet contained only three subgroups, which is lower than ~20 distinct lineages found in the empirical data. The main probable explanation for the discrepancy between simulations and observation is the simplified distance computation considered and the coarse resolution of the SDMs at 1° resolution used in our illustration. Genetic differentiation among populations might happen as a result of small scale landscape features that were not included in our reconstructed maps and distance matrices, such as small mountain ranges, rivers, forested areas, or other landscape features, which are influencing caribou population connectivity (Apps & McLellan, 2006; Avgar, Mosser, Brown, & Fryxell, 2013). More complex distance matrices should likely lead to increased lineage differentiation within each of the main genetic clusters. Furthermore, we used an illustration at coarse resolution for large spatial extent to run fast illustrative simulations of the method. Another limitation resulted from the accuracy of SDMs. A few specific isolated locations are either not suitable or not colonized in the simulations, which explain the lower complexity of simulated compared to observed genetic structure, as for instance in the case of Svalbard where a highly isolated population occurs (Yannic et al., 2018; Yannic, Pellissier, Ortego, et al., 2014). As a result, some lineages with significant differentiation are not reproduced in the simulations. To calibrate the SDMs, we used the IUCN range map rather than accurate locations of species observation. As such, the calibrated climatic niche of the species is not accurate and only captures the most obvious dimensions of the species ecological preferences. More accurate high resolution, but also computationally demanding simulations could generate more structured outputs with better agreement with the data. In the future, higher landscape complexity can be included in order to investigate whether with increased landscape features more complex genetic structure can emerge in the simulations, beyond the two main groups associated with the distinct refugees (Yannic, Pellissier, Ortego, et al., 2014).

### 5.1 Management implications and future directions

We provide a parsimonious simulation model that generates expectations on intraspecific genetic differentiation as a result of landscape dynamics illustrated with the case of the caribou/reindeer. We propose to simulate how the interactions between, (a) dispersal ability, (b) habitat suitability from SDMs, and (c) landscape connectivity shape the gene flow between populations and the generation of intraspecific structure over time. Our approach remains simple in nature and further properties can be considered. In particular, our approach simulates beta diversity, but not alpha diversity: considering continuous suitability as a proxy of carrying capacity within cells can provide the means to integrate all diversity facets within the same framework. In a future development, the strength of our framework could be reinforced by the use of ancient DNA that could, when available, simultaneously validate past distribution modeling as well as genetic lineage occurrences. Furthermore, the simulation model provides expectation for neutral genetic markers and it remains to be seen how processes can be integrated in a simplified form to study adaptive loci (e.g., Rebaudo, et al., 2013). The simulation model further allows forecasting species range shifts and genetic beta diversity under climate change scenarios and evaluates how species range shifts will induce genetic diversity erosion of species. García‐Díaz et al. (2019) show that quantitative models have already served an important role in generating effective conservation, policy, and management actions. We expect that a better understanding of the genetic changes as a result of past climate changes will help predict the future shifts of intraspecific genetic variation under ongoing climate changes.

## CONFLICT OF INTEREST

None declared.

## Supporting information

Supplementary Material

## Data Availability

Climate data are available from the CHELSA website (http://chelsa-climate.org/), and genetic data are available from the Dryad Digital Repository: http://dx.doi.org/10.5061/dryad.f971b
