# Supplementary figures and images for: Harnessing paleo‐environmental modeling and genetic data to predict intraspecific genetic structure
Source: Evol Appl. 2020 Jun 2;13(6):1526–42. doi: 10.1111/eva.12986 (PMC7359836; doi:10.1111/eva.12986)

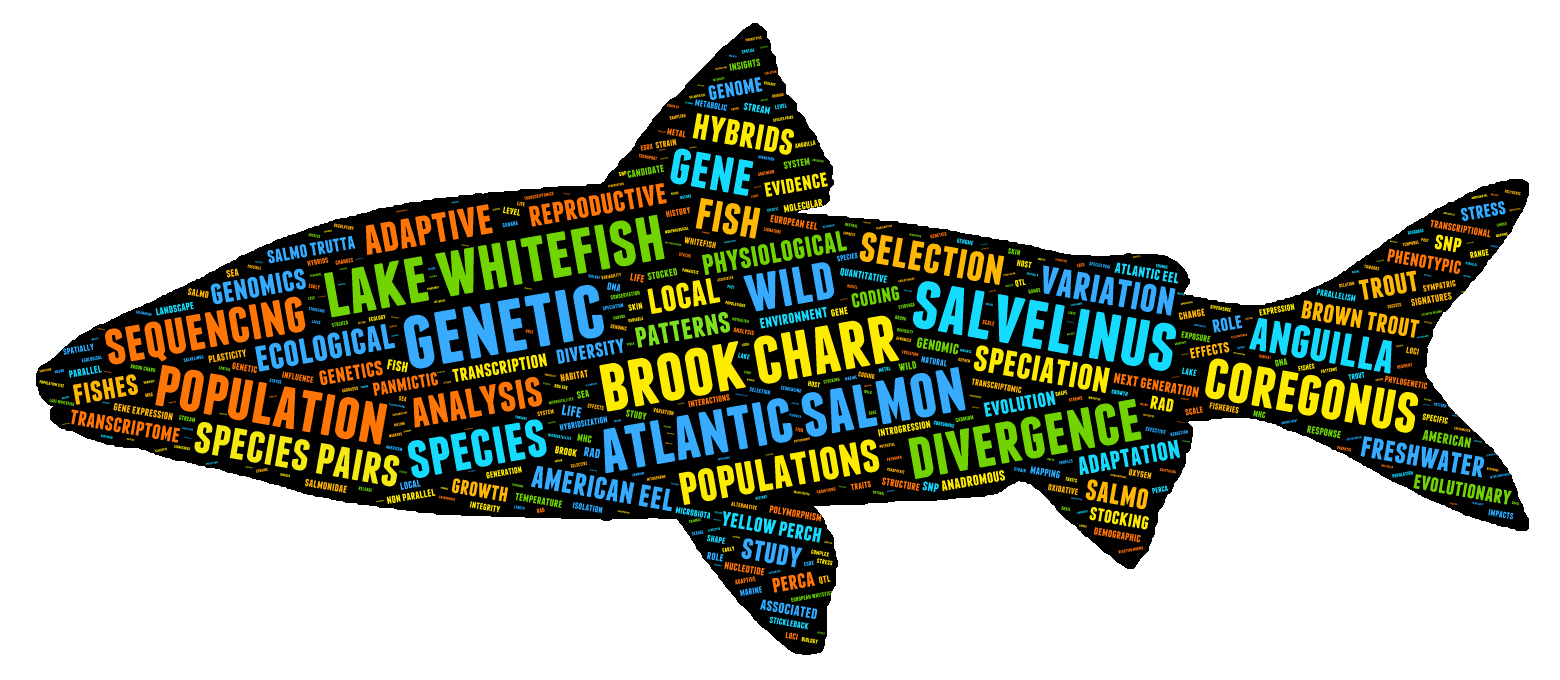

Supplement: Supplementary file 1 — Supplementary Material [file EVA-13-1526-s001.jpg]
